# Metabolomic and Proteomic Profiles Reveal the Dynamics of Primary Metabolism during Seed Development of Lotus (*Nelumbo nucifera*)

**DOI:** 10.3389/fpls.2016.00750

**Published:** 2016-06-07

**Authors:** Lei Wang, Jinlei Fu, Ming Li, Lena Fragner, Wolfram Weckwerth, Pingfang Yang

**Affiliations:** ^1^Department of Ecogenomics and Systems Biology, University of ViennaVienna, Austria; ^2^Key Laboratory of Plant Germplasm Enhancement and Speciality Agriculture, Wuhan Botanical Garden, Chinese Academy of SciencesWuhan, China; ^3^Vienna Metabolomics Center, University of ViennaVienna, Austria; ^4^Sino-African Joint Research Center, Chinese Academy of SciencesWuhan, China

**Keywords:** mass spectrometry, primary metabolism, GC-TOF-MS, LC-Orbitrap-MS, seed development, *Nelumbo nucifera*

## Abstract

Sacred lotus (*Nelumbo nucifera*) belongs to the Nelumbonaceae family. Its seeds are widely consumed in Asian countries as snacks or even medicine. Besides the market value, lotus seed also plays a crucial role in the lotus life cycle. Consequently, it is essential to gain a comprehensive understanding of the development of lotus seed. During its development, lotus seed undergoes cell division, expansion, reserve accumulation, desiccation, and maturation phases. We observed morphological and biochemical changes from 10 to 25 days after pollination (DAP) which corresponded to the reserve synthesis and accumulation phase. The volume of the seed expanded until 20 DAP with the color of the seed coat changing from yellow-green to dark green and gradually fading again. Starch and protein rapidly accumulated from 15 to 20 DAP. To further reveal metabolic adaptation, primary metabolites and proteins profiles were obtained using mass spectrometry based platforms. Metabolites and enzymes involved in sugar metabolism, glycolysis, TCA cycle and amino acid metabolism showed sequential dynamics enabling the clear separation of the different metabolic states during lotus seed development. The integration of the data revealed a highly significant metabolic switch at 15 DAP going through a transition of metabolically highly active tissue to the preparation of storage tissue. The results provide a reference data set for the evaluation of primary metabolism during lotus seed development.

## Introduction

Sacred lotus (*Nelumbo nucifera*) belongs to the small plant family of Nelumbonaceae (The Angiosperm Phylogeny Group, 2009), which is found at a basal position of eudicot plants and contains only one genus (*Nelumbo*) with only two species, sacred lotus and American lotus (*Nelumbo lutea*) (Pan et al., 2010). *N. nucifera* is distributed in Asia and Northern Australia, whereas *N. lutea* inhabits the eastern areas of North America and the northern area of South America. *N. nucifera* is a diploid (2*n* = 16) with an estimated 929 Mb genome (Ming et al., 2013). In Asia, sacred lotus is an economically important crop with a very long cultivation history. It is widely used as food, medicine, and ornamental plant. Based on its agricultural and horticultural utilization, it can be divided into three groups, namely flower, seed, and rhizome lotus.

The seed plays an important role in plants life cycle. It stores genetic information and nutrients and also develops protective and defense strategies to guarantee the reproduction of the next generation. Sacred lotus seeds are the products of its sexual reproduction, and primarily used to reproduce and distribute its progeny. It has been reported that lotus seeds have an extreme longevity, and can remain viable for about 1300 years (Shen-Miller, 2002). The stored carbohydrates, proteins, lipids and other compounds not only provide energy for seed germination but also for human and other animals in the form of food. The seeds of sacred lotus are widely consumed in Asian countries as snacks or in some cultures for medicinal purposes (Yen et al., 2005, 2006). Sacred lotus blossoms and sets seeds in the hot summer days, which makes its seed development responsive to high temperatures. Based on this economic importance, it is important to study its seed formation and development.

Carbohydrates, proteins and oils are three major reserves accumulating in plant seeds (Weber et al., 2005). Compared to other plants, sacred lotus seeds mainly accumulate starch, which accounts for about 60% of its total dry weight. It also accumulates about 8% protein in immature seeds and as high as 24% in the mature desiccated seeds (Zheng et al., 2003; Bhat and Sridhar, 2008). In contrast to cereals, starch is mainly synthesized and accumulated in cotyledons in sacred lotus. Previous studies on lotus seed was mainly focused on the identification of its nutritional constituents and medicinal components (Yen et al., 2005, 2006; Mukherjee et al., 2010). However, there is thus far no report exploring the regulation of biosynthesis and accumulation of these reserves. Because the accumulation of reserves is important in both nutrition and in an economical sense, studies on seed filling have been widely conducted in various of crops, including rice (Xu et al., 2008), barley (Finnie et al., 2002), wheat (Laino et al., 2010), maize (Mechin et al., 2007), soybean (Agrawal et al., 2008), oilseeds (Hajduch et al., 2005, 2006), and *Medicago truncatula* (Gallardo et al., 2003, 2007; Repetto et al., 2008). All these studies revealed that the seeds experienced dramatic changes in morphology and metabolism. In contrast to other crops, sacred lotus seeds can be consumed either freshly or in a mature desiccated form. As fresh food, sacred lotus seed is sweet, which indicates it contains high contents of soluble sugars. After this, it enters the filling stage, during which starch is quickly synthesized and accumulated. When consumed maturely, the seeds are desiccated and cotyledons are filled with starch. Understanding when this transition from the stage suitable for fresh consumption to the filling stage and how this transition happens, are very important questions in sacred lotus seed production. With the development of sequencing technologies, great success has been achieved in genome sequencing. Recently, the sacred lotus whole genome was sequenced (Ming et al., 2013), which has provided ample information for the analysis of the transcriptome and proteome of this species (Deng et al., 2015; Yang et al., 2015a,b; Liu et al., 2016). Moro et al. (2015b) profiled the endosperm proteome of mature lotus seed and they also compared the proteome profiles of the immature and mature seed endosperm. However, these data did not provide a clear answer on the transition from the fresh consumption stage to the reserves filling stage. With this in mind, we combined a label-free quantitative proteomics and gas-chromatography-mass spectrometry (GC-MS) based metabolomic studies on the developing sacred lotus seeds in an effort to address this question. The first objective is to identify key enzymes important for sacred lotus seed development and reserve filling. The second is to reveal the metabolic dynamics during seed development, and the third is to shed light on the mechanisms underlying the switches in metabolism during sacred seed development and maturation.

## Materials and Methods

### Plant Material

The sacred lotus (*N. nucifera*) cultivar “Baijianlian” were cultivated in the experimental field of Wuhan Botanical Garden (30°32′45″N 114°24′52″E), Chinese Academy of Sciences in late April. In July, most of the sacred lotus plants blossomed. The healthy plants were selected for experiments. In the first day of blossoming, the flowers were manually pollinated and capped with labeled paper bags. Seeds at different days-after-pollination (DAP) were harvested, and then either fixed in formalin-acetic acid-alcohol (FAA) solution for microscopy analysis or frozen with liquid nitrogen and stored at -80°C for metabolite and protein extraction.

### Histological Procedures

The harvested samples were fixed in FAA solution for at least 24 h, and then vacuum-dried for 1 h. Thereafter, the samples were subjected to sequential dehydration with 30–100% ethanol, and then embedded into paraffin. The embedded samples were cut into 6–10 μm slices using a LEICA2150 rotary microtome (Leica, German). After dewaxing, the slices were subjected to Periodic Acid Schiff (PAS) solution and Coomassie Brilliant Blue (CBB) staining for the observation of starch and protein accumulation, respectively. An Olympus BX-61 upright metallurgical microscope (Olympus, Japan) was used for the observation.

### Metabolite Extraction and Analysis

The harvested lotus seeds were ground to powder in liquid nitrogen with a mortar and pestle. 100 mg material was extracted with 1.4 ml precooled methanol. The extraction mixture was homogenized by vortexing for 10 s. 60 μl of ribitol (0.2 mg ml^-1^) was added to the mixture as internal standard followed by 10 s of vortexing. This solution was ultrasonicated for 10 min, followed by centrifugation at 11 000 g for 10 min. The supernatant was transferred to a new tube and mixed with 750 μl of precooled chloroform and 1.5 ml of precooled water. The mixture was then centrifuged at 2200 g for 15 min. 150 μl of extraction solution from upper phase was dried under vacuum and stored at -80°C until derivatisation.

The extract of lotus seed was methoxyaminated and silylated according to the derivatizatation method from Weckwerth et al. (2004) with minor modifications. The dried extract was dissolved in 20 μl of methoxyamine hydrochloride pyridine solution (40 mg ml^-1^) and incubated at 30°C with vigorous shaking for 90 min. Before silylation, 30 μl of even-numbered alkanes (C10–C40, 50 mg ml^-1^ each, Fluka, Germany) was spiked in 1 ml of *N*-methyl-*N*-(trimethylsilyl) trifluoroacetamide (MSTFA, MACHEREY-NAGEL, Germany) solution. Then 80 μl of this MSTFA-Alkane solution was added to the sample followed by 30 min incubation at 37°C with vigorous shaking. The derivatized sample was centrifuged at 20 000 *g* for 8 min. The supernatant was then transferred to vials for measurement.

Samples were measured with an Agilent 6890 gas chromatography coupled to a LECO Pegasus^®^ 4D GC × GC-TOF spectrometry (GC-TOF-MS). Instrument parameter settings were consistent with a previous report (Doerfler et al., 2013). Each sample was injected under both splitless and split 25 times mode for better quantification of candidates with a wide capacity range.

The obtained raw files were deconvoluted with LECO Chroma TOF^®^. The retention times (RTs) of alkanes were applied to calibrate the RTs of candidates. Candidates were manually annotated by comparing their RTs and mass spectra to those of standards in GMD database (Kopka et al., 2005) with a minimum match factor of 700. Peak areas of annotated candidates corresponding to specific masses were integrated and used for relative quantification. The peak areas of the same metabolite with different derived groups were merged and normalized to internal standard ribitol.

### Protein Extraction and Analysis

Proteins were extracted using the phenol method according to previously description (Liu et al., 2013). Briefly, the samples were ground to power with liquid nitrogen, homogenized in pre-cooled homogenization buffer (20 mM Tris/HCl (pH 7.5), 250 mM sucrose,10 mM ethylene glycol-bis(b-aminoethylether)-N,N,N′,N′-tetraacetic acid (EGTA), 1 mM PMSF, 1 mM DTT, and 1% Triton X-100), followed by centrifugation at 12 000 *g* for 20 min at 4°C. Supernatants were collected, and mixed with equal volume of Tris-phenol (pH > 8.0) by vortexing, and centrifuged again. The top phenol phase was carefully transferred to a new tube, and mixed with 5 volumes of 0.1 M methanolic ammonium acetate in 100% methanol, then incubated overnight at -20°C. The precipitated proteins were washed one time with 0.1 M methanolic ammonium acetate and two times with acetone. The protein pellets were vacuum-dried and then stored at -80°C for further use.

For one-dimensional electrophoresis (DE), proteins were dissolved in lysis buffer (7 M urea, 2 M thiourea, 4% w/v CHAPS, and 65 mM DTT) and quantified using the Bradford method (Bradford, 1976). Twenty-five micro gram of total proteins were loaded onto 12.5% sodium dodecyl sulfate–polyacrylamide gel (SDS-PAGE) and run at 50 V until bromophenol blue line enter into the resolving gel for about 1 cm. After running, the gels were stained with (CBB R-250) solution, and then destained until protein bands were clearly visualized. The gel was cut into small slices, and subjected to in-gel trypsin digestion (Liu et al., 2016).

For in-solution digestion, 100 μg of protein was reduced with 5 mM dithiothreitol (DTT) at 37°C for 45 min then alkylated with 10 mM iodoacetamide (IAA) at 23°C in dark for 60 min. Finally the reaction was finished with addition of 5 mM of DTT (23°C, dark, 15 min). Endoproteinase LysC and trypsin were applied for digestion based on the protocol reported before (Hoehenwarter et al., 2008).

Peptides from each sample were thoroughly dissolved in 4% acetonitrile and 0.1% formic acid, and desalted. Afterward, 10 μg peptides sample was subjected to analysis using nanoHPLC coupled to LTQ-Orbitrap-MS according to previously report (Liu et al., 2016). MS analysis was performed on an Orbitrap LTQ XL mass spectrometer (Thermo, Germany) with a controlled flow rate of 500 nl min^-1^. Specific tune settings were 1.8 kV for spray voltage and 180°C for temperature of the heated transfer capillary. MS raw data were searched using the SEQUEST algorithm from Proteome Discoverer version 1.3 (Thermo, Germany). The filter criteria were set as follows. (I) 1% false discovery rate (FDR); (II) a variable modification of acetylation of N-terminus and oxidation of methionine; (III) mass tolerance of 10 ppm for parent ion and 0.8 Da for fragment ion.

The MS data were searched against the newly annotated genome database of lotus^1^ to identify proteins. The assembled genome of lotus was 804 Mb, representing 86.5% of the estimated lotus genome. Following repeat-masking and annotation, 26,685 unigenes were inferred, 82% of them have similarity to proteins in SwissProt (Ming et al., 2013). The searching parameters are as follows: (i) medium or high peptide confidence; (ii) minimum Xcorr of 1 for +1, 2 for +2 and 3 for +3 charged peptides, etc. Subsequently, a label-free approach based on spectral counts (SpCs) was used to quantify the identified proteins followed by a normalized spectral abundance factor (NSAF) normalization strategy (Zybailov et al., 2006). The NSAF was calculated based on the formula: ${NSAF}_{i}=({SpC}_{i}/L_{i})/\Sigma_{i=1}^{N}(SpC/L)i$. The NSAF for a protein is the number of spectral counts (SpCs) divided by protein length (L) and then divided by the sum of SpC/L. The obtained raw files and sequence information of the identified proteins were submitted to the public repository ProteomeXchange (Vizcaino et al., 2014) with the dataset identifier PXD003768^2^ (Username: reviewer72127@ebi.ac.uk, Password: LHImleH6). Furthermore all lotus proteomic data, annotated peptide spectra and experimental meta information is stored at the proteomics database PROMEX^3^ (Hummel et al., 2007; Wienkoop et al., 2012).

### Multivariate Statistical Analysis

Principal component analysis (PCA) and hierarchical clustering analysis were performed with the statistical tool box COVAIN (Sun and Weckwerth, 2012). Analysis of variance (one-way ANOVA) was performed with SPSS Software Package and the significant levels were indicated with lower case letters according to Duncan’s test (*p* < 0.05). Duncan’s test was done in SPSS with an option in *post hoc* window named “Duncan” at 0.05 level. Venn diagram was performed with online free software Venny 2.1^4^.

## Results and Discussion

### Morphological Changes during Lotus Seed Development

**FIGURE 1:**
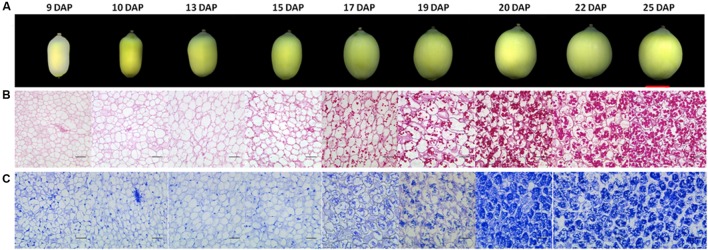
**Morphology and histology of developing lotus seeds. (A)** Lotus seed at different developmental stages. Histological observation of polysaccharides **(B)** and protein accumulation **(C)** during lotus seed development. The polysaccharides and proteins were stained with PAS and Commassie blue method, respectively. The rulers indicate 1 cm in **(A)** whereas 100 μm in **(B,C)**.

It has been reported that the development of lotus seed could be divided into four phases, phase I cell division and organ formation (1–3 days after pollination, DAP), phase II cell enlargement (4–9 DAP), phase III reserves synthesis and accumulation (10–25 DAP), phase IV desiccation and maturation (Tang et al., 1989). Based on the objectives of this study, we focused on phase III. Sacred lotus seeds at different time points of this phase were harvested with three biological replicates. For comparison, seeds at 9 DAP were also collected. The size of lotus seeds expanded gradually until 20 DAP, and then slowed down (**Figure 1A**). The seed coat color gradually changed from yellow-green to dark-green until 17 DAP, and then gradually faded (**Figure 1A**). To further reveal the accumulation of different reserves, light microscope observations were applied on the cotyledons of these samples. No starch accumulation was observed before 10 DAP, whereas starch accumulated rapidly after 15 DAP, and the accumulation rate slowed after 20 DAP (**Figure 1B**). Proteins accumulated in a similar pattern (**Figure 1C**). Interestingly, clear nuclei could be observed in the cells before 15 DAP, while this signal disappeared after 15 DAP, which might be correlated with the accumulation of starch and proteins. Based on these data, we selected the cotyledons at 10, 15, 20, and 25 DAP for further metabolomic and proteomic studies in order to explore the metabolic switch during this phase.

### Metabolomic Profile of Developing Lotus Seed

**FIGURE 2:**
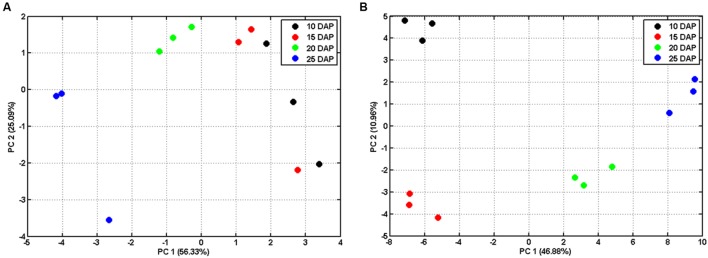
**Principal component analysis (PCA) of metabolomic (A) and proteomic datasets (B) during lotus seed development.** The normalized metabolite dataset (Supplementary Table S1) and NSAFs of all the identified proteins (Supplementary Table S3 sheet “Combined”) were log 10 transformed and used for PCA. The NSAFs of those proteins that identified with both in-gel and in-solution procedures were averaged for PCA.

**FIGURE 3:**
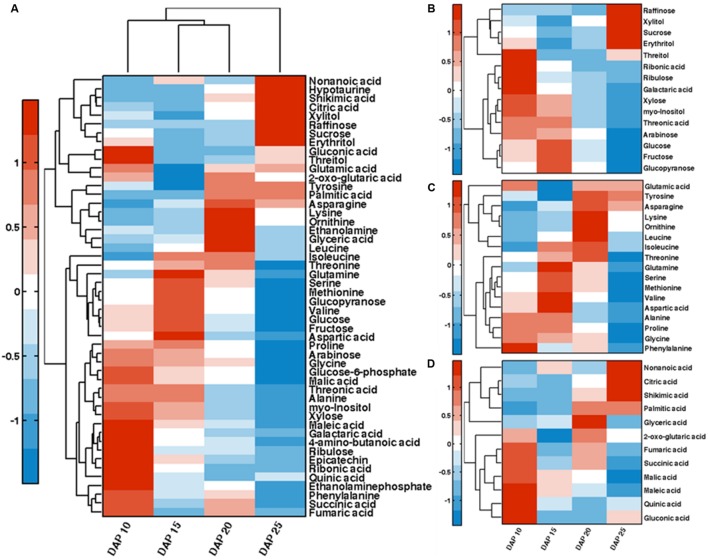
**Bi-clustering analysis of annotated primary metabolites (A).** Clustering analysis of the dynamics of sugars **(B)**, amino acids **(C)**, and organic acids **(D)** during lotus seed development.

In order to explore the dynamic changes of primary metabolites such as sugars and organic acids, we analyzed metabolites of developing lotus seed. The extracted metabolites were derivatized before measuring by gas chromatography-time of flight-mass spectrometry (GC-TOF-MS). In total, 95 candidates were annotated and quantified based on their spectra and retention times (Supplementary Table S1). Bi-cluster analysis of the metabolites presented distinctive metabolic profiles of lotus seed at different developmental stages with the changing patterns of the metabolites shown in a hierarchical heat map (**Figure 3A**). Samples at 25 DAP were most distinctive compared to samples at other stages. PCA plots of the metabolite data show the trajectory of lotus seed development (**Figure 2A**). Samples of 10 and 15 DAP are separated from those of 20 and 25 DAP on PC 1, which accounts for over 50% of the separation. Loading values in Supplementary Table S2 indicate sugars e.g. glucose, fructose, xylose, *myo*-inositol contribute greatly to this separation. Fructose and glucose, the main sugars in lotus seed, with peak accumulation at 15 DAP, notably contributed to the separation of developmental samples at the first three major PCs. Other sugars, e.g., ribulose, *myo*-inositol, glucose-6-phosphate, arabinose, and xylose were most abundant at 10 DAP and decreased during lotus seed development. Raffinose, xylitol, sucrose, and erythritol greatly accumulated in seeds at 25 DAP (**Figure 3B**).

Amino acids like alanine, glutamine and lysine were also essential contributors to the characterization of developmental stages. Most of the amino acids detected showed highest level at 15 DAP or 20 DAP except phenylalanine, glycine, and glutamic acid, which were highest at 10 DAP (**Figure 3C**). 10 out of 17 detected amino acids showed lowest level at 25 DAP, which may have resulted from the incorporation of amino acids into storage protein synthesis. This is consistent with the histological observations (**Figure 1C**) of considerable protein accumulation at 25 DAP.

Organic acids are another class of metabolites involved in essential aspects of metabolism in plants. The predominant organic acid in lotus seed is malic acid which decreased in phase III. 2-oxo-glutarate (also known as α-ketoglutarate), succinate and fumarate involved in TCA cycle presented similar fluctuating pattern. These three metabolites decreased from 10 to 15 DAP followed by an increase and another decrease during lotus seed development (**Figure 3D**). Citric acid was the only TCA cycle intermediate that significantly increased from 10 to 25 DAP. Shikimic acid, the precursor for aromatic amino acids also increased notably at this developmental phase.

### Proteomic Profile of Developing Lotus Seed

**FIGURE 4:**
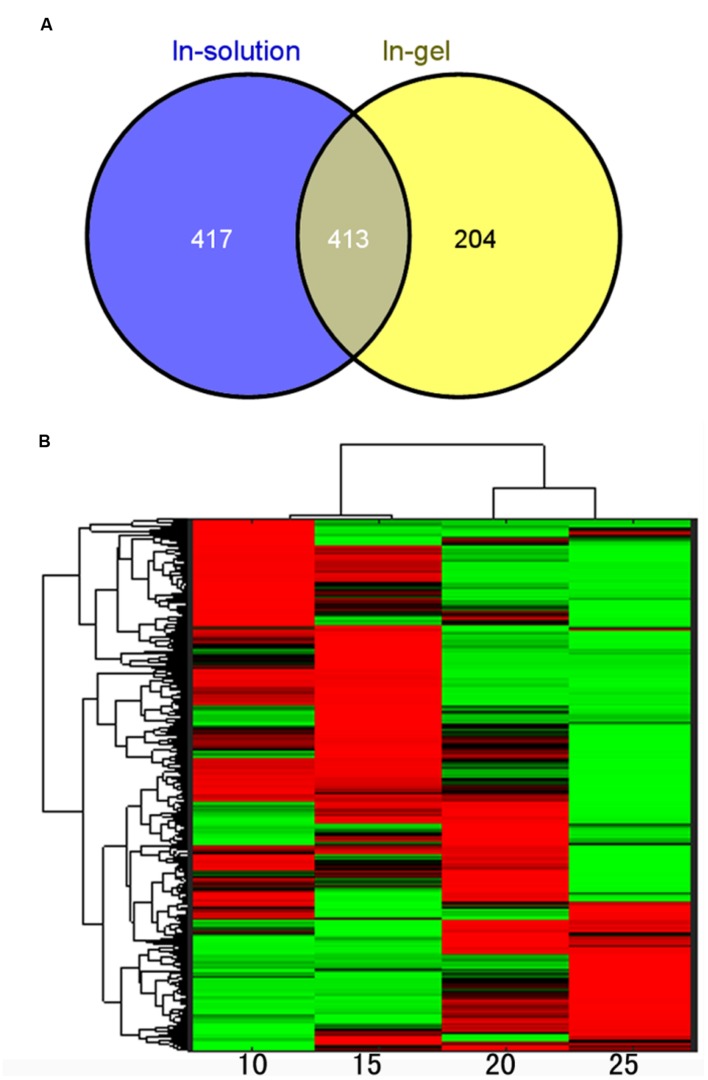
**Quantitative analysis of the proteomic data. (A)** Venn diagram showing the overlap of proteins identified in samples using in-gel and in-solution digestion methods; **(B)** Clustering analysis of the identified proteins.

To further understand the molecular mechanisms underlying the dynamic changes of metabolites and the shift from physiological active status to reserves accumulation, comparative proteomic analysis was conducted on the developing lotus seeds at 10, 15, 20 and 25 DAP. In order to obtain as much information as possible, an in-gel digestion via LC-MS/MS strategy was conducted in parallel with an in-solution digestion strategy. Based on the criteria described in the Methods, a total of 1140 and 1610 proteins were identified through in-gel (Valledor and Weckwerth, 2014) and in-solution digestion techniques, respectively, among which 617 and 830 proteins were quantified, respectively, (Supplementary Table S3). The number of identified proteins is much more than that from the other groups working with lotus (Moro et al., 2015a,b). The reasons might be as follows. Firstly, samples from more stages were used in this study. Secondly, the MS data were searched against a self-generated lotus protein database in this study rather than that available in NCBI database, which will increase the rate of successful protein identifications. However, there are 36 proteins that were previously identified by 1-D and 10 by 2-D (Moro et al., 2015a,b) methods that were not identified in this study (Supplementary Table S4). This might because the lotus genome is not well annotated, which will result in the loss of some information for protein identifications. Furthermore, we used the seeds including embryos and endosperms, which may result in the loss of some embryo specific proteins. Among all the annotated protein candidates, 413 proteins were commonly identified and quantified in both strategies (**Figure 4A**). To evaluate the reproducibility of biological replicates, PCA was performed as previously reported (Liu et al., 2016) with all the identified proteins. The PCA plot shows clear separation between samples from different developmental stages (**Figure 2B**). Specifically, samples from 10 and 15 DAP were not separated by PC1 which accounts for 46.88%, but by PC2 accounting for 10.96% (**Figure 2B**), which is similar to the PCA result from the metabolomic data. Interestingly, clustering analysis based on the expressional patterns of all the quantified proteins also shows that samples of 10 and 15 DAP were separated from those of 20 and 25 DAP (**Figure 4B**). These data indicate that seeds are metabolically active at the first two stages, and enter into reserve accumulation at the latter two stages.

**FIGURE 5:**
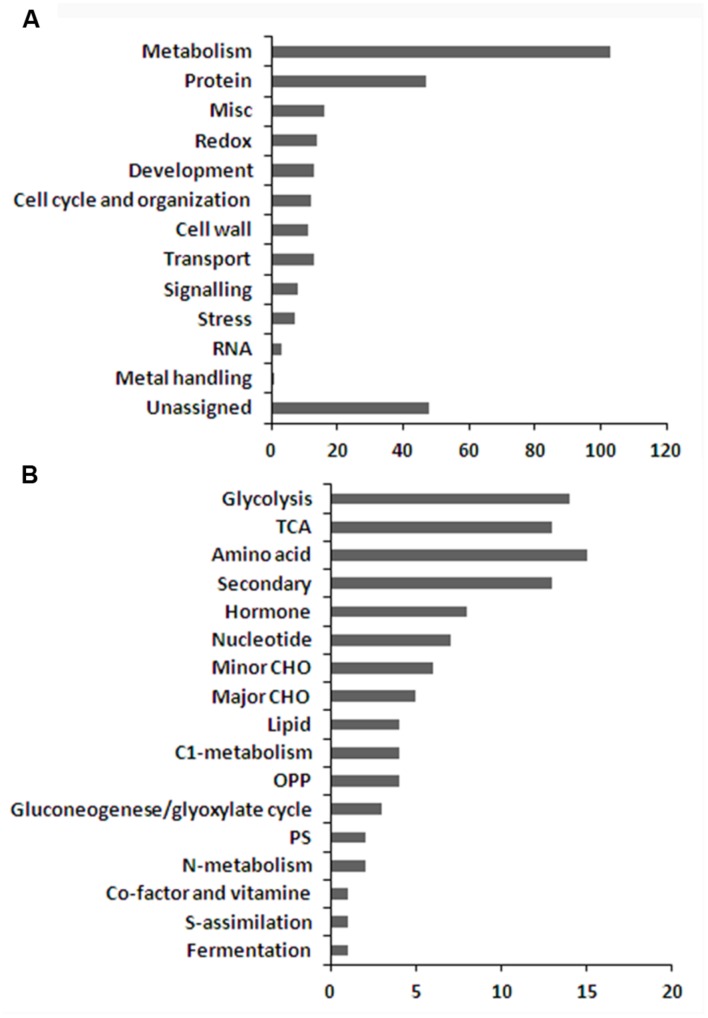
**Functional categorization of the differentially displayed proteins (A,B) sub-functional categorization of the metabolism related proteins**.

Quantitative analysis showed that 296 proteins were significantly changed during seed development (Supplementary Table S5). Except for 48 function-not-assigned proteins, the rest of the proteins were affiliated to 12 functional groups based on MapMan descriptions (**Figure 5A**). 103 out of 296 significantly changed protein candidates, which accounts for about 34.8%, are metabolism related proteins indicating that the seeds are subject to dramatic metabolic changes during these developmental stages. These proteins could be further classified into 17 sub-groups (**Figure 5B**), including glycolysis, TCA, OPP, gluconeogenese/glyoxylate cycle, photosynthesis, S-assimilation, fermentation, and metabolism of amino acids, secondary metabolites, hormone, nucleotides, minor carbohydrates (CHO), major CHO, lipids, C1-, nitrogen, co-factor, and vitamin.

### Integration of Metabolomic and Proteomic Profiles to Reveal Primary Metabolism Processes during Lotus Seed Development

**FIGURE 6:**
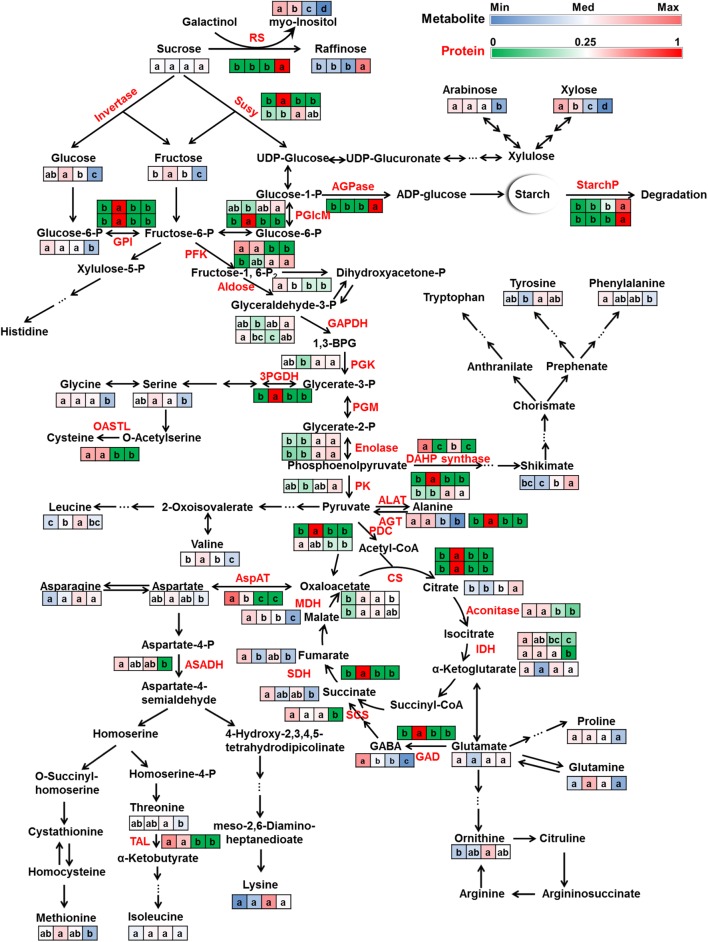
**Primary metabolism dynamics during lotus seed development**. Metabolites (black) and proteins (red) involved in sugar metabolism, glycolysis, TCA cycle and amino acid metabolism were mapped on their metabolism pathways. The relative ratios of normalized metabolite peak areas were colored with blue-white-red color bar corresponding to the values from the minimum (Min) to median (Med) to maximum (Max). The relative ratios of the protein NSAFs were colored with green-white-red color bar corresponding to the ratios from 0 to 0.25 to 1. All the protein candidates that annotated as the same enzyme were list here in consecutive rows. Four squares from left to right indicate samples of 10, 15, 20, and 25 DAP, respectively. Lowercase characters in the squares indicate significant levels according to Duncan’s test (*p* < 0.05). Abbreviations: RS, raffinose synthase; Susy, sucrose synthase; AGPase, ADP glucose pyrophosphorylase; StarchP, starch phosphorylase; PGlcM, phosphoglucomutase (plastid); GPI, glucose-6-phosphate isomerase (plastid); PFK, phosphofructokinase; GAPDH, glyceraldehyde 3-phosphate dehydrogenase; 1,3-BPG, 1,3-Bisphosphoglyceric acid; PGK, 3-phosphoglycerate kinase; PGM, phosphoglycerate mutase; PK, pyruvate kinase; PDC, pyruvate dehydrogenase complex; CS, citrate synthase; IDH, isocitrate dehydrogenase; SCS, succinyl coenzyme A synthetase; SDH, succinate dehydrogenase; MDH, malate dehydrogenase; GAD, glutamic acid decarboxylase; GABA, 4-aminobutanoic acid (γ-Aminobutyric acid); AspAT, aspartate aminotransferase; ALAT, alanine aminotransferase; AGT, alanine-glyoxylate aminotransferase; ASADH, aspartate semialdehyde dehydrogenase; TAL, threonine ammonia-lyase; 3PGDH, 3-phosphoglycerate dehydrogenase; OASTL, *O*-acetylserine(thiol)lyase; DAHP synthase, 3-deoxy-D-arabino-heptulosonate 7-phosphate synthase.

The seed of lotus, as a sink organ, plays a central role in the lifecycle of lotus. Primary metabolism plays an essential role in plant metabolism and is vital for the plant to survive and develop. Thus, the understanding of primary metabolism of the seed is crucial. The morphological description of the processes alone is not sufficient to interpret such a complex process. Here we explored metabolic regulation and adjustments during this process at the molecular level by integrating metabolomic and proteomic profiles of the developing seeds of lotus (**Figure 6**). Central primary metabolism pathways including sugar metabolism, glycolysis, TCA cycle, and amino acids metabolism are discussed below.

#### Sugar Metabolism

The predominant sugars in lotus seed include sucrose, glucose, and fructose. Sucrose is mainly produced in leaves via photosynthesis and then transported to seeds and other organs for energy and carbon skeleton production. At early developmental stages, the seed embryo undergoes fast cell division and expansion. During this period, the transported sucrose is predominantly hydrolyzed to glucose and fructose by sucrose synthase and invertase which results in an increase of these hexoses. The incorporation of these hexoses into glycolysis and TCA cycle provides energy and precursors for seed growth. Since 20 DAP, the volume expansion of the seed slowed down and the seed moved into its maturation phase. At this stage, sucrose metabolism, instead of mainly hydrolyzing to hexoses, switched to raffinose synthesis and resulted in significant accumulation of raffinose in mature seeds as well as significant reductions of glucose and fructose. The accumulated raffinose serves as an energy and carbon storage reserve as well as protectants (Peterbauer and Richter, 2001; Karner et al., 2004) for further germination and against stresses. Enzymes involved in these processes, e.g., sucrose synthase (Susy), raffinose synthase (RS) showed consistent changing patterns with these metabolites. Susy, which catalyzes the reversible conversion of sucrose to fructose and UDP-glucose, showed highest level at 15 and 20 DAP while RS was highest in mature seeds (**Figure 6**). Invertase, which was reported to be crucial for carbohydrate metabolism during seed development (Weber et al., 1997; Sturm, 1999) was not detected. Sucrose is either converted to hexoses or to larger polymers such as raffinose or starch. ADP glucose pyrophosphorylase (AGPase), the key enzyme in the starch biosynthetic pathway (Sakulsingharoj et al., 2004; Weigelt et al., 2009), was highly abundant at 25 DAP (**Figure 6**). Previous studies indicated elevated cytoplasmic AGPase activity resulted in increased flux of sucrose into starch (Sakulsingharoj et al., 2004) whereas AGPase repression stimulated glycolytic and TCA cycle metabolism (Weigelt et al., 2009). In the present study, starch accumulation was accompanied with a high expression of AGPase, which is consistent with these reports. Starch phosphorylases (StarchP), which was reported to be associated with initiating seed reserve starch accumulation in endosperm (plastidic StarchP) and starch degradation (cytosolic StarchP) during germination (Schupp and Ziegler, 2004), also significantly accumulated in mature lotus seed. During seed development, sugar composition and concentration highly varied to accomplish different steady states of metabolism. Such sugar partitioning was also observed in other developing seeds, e.g., *Arabidopsis* (Fait et al., 2006), barley (LaBerge et al., 1973), and wheat (Schupp and Ziegler, 2004). From 10 to 25 DAP, sucrose content slightly increased. The relatively constant sucrose level indicated a balance between sucrose import and consumption.

#### Glycolysis

Glucose and fructose further metabolize along the glycolytic pathway to produce energy (ATP), reductant (NADH) and pyruvate production. The consumption together with the aforementioned reduced production of these hexoses caused a decrease of glucose and fructose after 15 DAP. In the present study, enzymes involved in seven steps, i.e., glucose-6-phosphate isomerase (GPI), phosphofructokinase (PFK), aldose, glyceraldehyde 3-phosphate dehydrogenase (GAPDH), 3-phosphoglycerate kinase (PGK), enolase, and pyruvate kinase (PK) changed significantly (**Figure 6**) indicating momentous adjustment of glycolytic metabolism during lotus seed development. These enzymes did not change with identical patterns, e.g., aldose was highest at 10 DAP, GPI 15 DAP whereas PGK, enolase and PK accumulated at 20 DAP and/or 25 DAP. One of the reasons might be related with the “bottom up” regulation of plant glycolysis (Plaxton, 1996), since glycolysis also plays a biosynthetic role in plants, especially in actively growing tissues. The intermediates generated during glycolysis are precursors for amino acids, fatty acids (Qi et al., 1995; Schwender et al., 2015), and other secondary metabolites. The incorporation of the intermediates to other anabolic pathways caused the fluctuation of these substances and a feedback to the system. Enzyme accumulation then adjusted based on these feedback signals and caused various changing patterns of enzyme capacities. Another point to be considered is that posttranscriptional modification can also regulate metabolism by enhancing or attenuating enzyme activity (Daran-Lapujade et al., 2007; Oliveira and Sauer, 2012; Tripodi et al., 2015). This means enzyme capacity is not a unique parameter that affects metabolic regulation. In future studies, enzyme activity information should be complemented with the protein abundance data.

#### TCA Cycle

The TCA cycle operates as a bridge that connects carbohydrate, amino acid, lipid, and protein metabolism and also as an engine that generates energy and reductive power to drive metabolism, especially in sink organs. During lotus seed development, the intermediates were most abundance at 10 DAP and lowest in mature seed, except citrate which increased during seed ripening. Enzymes involved in the TCA cycle showed their lowest level in mature seed, except malate dehydrogenase (MDH) which was lowest at 10 DAP but also decreased from 15 to 25 DAP. The reduction of metabolic capacity of the TCA cycle at both the metabolic and proteomic levels indicated a decrease in energy, reducing power, and carbon skeleton generation during lotus seed development. This reduction in TCA cycle flux during seed development was also observed in developing *Arabidopsis* seed (Fait et al., 2006). One reason could be the shortage of oxygen, which is required by respiration to generate ATP and reducing equivalents by consuming ADP and reducing agents (Fait et al., 2006; Araujo et al., 2012). Another reason might be the decreasing requirement of energy and precursors from the TCA cycle as the seed at 25 DAP accomplished storage nutrition accumulation and prepared to enter the dormancy stage.

#### Amino Acid Metabolism

Amino acid metabolism is closely connected with the glycolytic pathway and the TCA cycle. On the one hand, intermediates generated along glycolysis and the TCA cycle can be precursors for amino acid synthesis. Nitrogen assimilation commonly happens in leaves, the biosynthesized amino acids are transported to sink organs, e.g., seed. Amino acids further serve as basic building blocks for protein synthesis and precursors for lipids and secondary metabolites, i.e., flavonoids, terpenoids, and alkaloid synthesis. At 25 DAP, the reduction of most of the amino acids might due to the incorporation of the amino acids into storage protein synthesis. On the other hand, amino acids can be catabolized and incorporate into glycolysis and TCA cycle for energy status maintenance (Angelovici et al., 2011; Galili et al., 2014; Hildebrandt et al., 2015). Lysine, a member of aspartate family, was reported to negatively correlate with TCA cycle activity in developing *Arabidopsis* seed which might cause energy shortage for seed development and germination in high lysine mutants (Angelovici et al., 2011). Junker and coworkers observed an redirection of fluxes leading into and out of the TCA cycle when cultivating *Brassica napus* embryos on a medium with ammonium nitrate as inorganic nitrogen source comparing with those with glutamine and alanine as organic nitrogen source (Junker et al., 2007). Their results indicated the effect of amino acids on metabolic flux. In summary, amino acid metabolism is affected and/or regulated by diverse factors, e.g., transportation, synthesis, and catabolism, together with interactions with other metabolic pathways.

## Conclusion

Previous studies divided the development of lotus seed into four phases with phase III (10–25 DAP) being defined as the stage of reserve synthesis and accumulation (Tang et al., 1989). Based on our microscopic observations, there was a very clear switch in the metabolic status in lotus seed cotyledons during this phase. As mentioned above, sacred lotus seeds can be consumed either freshly or in a mature desiccated form, which is determined by the transition from the stage containing a high content of soluble sugars to a high starch content and desiccated stage. To know when this transition occurs is important for lotus seed production, and this information can provide guidelines, especially for fresh seed consumption. Integration of metabolome and proteome dynamics indicated that lotus seed was still metabolically from 10 to 20 DAP. Peak accumulation of soluble sugars at 15 DAP together with the significant accumulation of starch at 20 DAP suggests lotus seeds at the time of 15–20 DAP with a relatively high sweetness are most suitable for fresh consumption. Central primary metabolism switched from an energetic mode to a storage mode with an accumulation of starch and protein at 15 to 17 DAP. After 20 DAP, seed volume slowed and lotus seeds reached their final size. Soluble sugars decreased whereas starch and protein accumulated at the end of phase III when lotus seed turned to a storage tissue. The mature lotus seeds with high content of starch and protein are more suitable for drying and snack production or to be used in some other cuisine. This study provides solid evidence outlining the shift in metabolic pathways during lotus seed filling, and also clear clues for the harvesting of sacred lotus seeds as a fleshy fruit.

## Author Contributions

PY and WW conceived and designed the experiments. LW, JF, LF, and PY performed the experiments. LW, PY, and ML analyzed the data. WW and PY provided the reagents, materials, and analytical tools. LW and PY wrote the manuscript. All the authors approved the final manuscript.

## Conflict of Interest Statement

The authors declare that the research was conducted in the absence of any commercial or financial relationships that could be construed as a potential conflict of interest.

## Footnotes

1 http://lotus-db.wbgcas.cn

2 http://www.proteomexchange.org

3 http://promex.pph.univie.ac.at/promex/

4 http://bioinfogp.cnb.csic.es/tools/venny/index.html
