# Supplementary material for: Metabolomic and Proteomic Profiles Reveal the Dynamics of Primary Metabolism during Seed Development of Lotus (Nelumbo nucifera)
Source: Front Plant Sci. 2016 Jun 7;7:750. doi: 10.3389/fpls.2016.00750 (PMC4894879; doi:10.3389/fpls.2016.00750)
Supplement: Supplementary file 4 [file Data_Sheet_4.DOCX]

**Table S4** Proteins that were only identified in previous studies in lotus seeds

| Protein description | Tissue | Methods |
| --- | --- | --- |
| 5-methyltetrahydrop teroyltriglutamate–homocysteine methyltransferase | Mature endosperm, embryo | 1-D |
| Heat shock protein 81-1 | Mature endosperm, embryo | 1-D |
| Cell division control protein 48 homolog D | Mature endosperm, embryo | 1-D |
| Heat shock protein 82 | Mature endosperm | 1-D |
| Eukaryotic initiation factor 4A-1 | Mature endosperm | 1-D |
| UTP–glucose-1-phosphate uridylyltransferase 1 | Mature endosperm | 1-D |
| Alpha-1,4 glucan phosphorylase L isozyme | Mature endosperm | 1-D |
| Fructose-bisphosphate aldolase, cytoplasmic | Mature endosperm, embryo | 1-D |
| Probable2,3-bisphosphoglycerate-independent phosphoglycerate mutase | Mature endosperm | 1-D |
| Granule-bound starch synthase 1 | Mature endosperm | 1-D |
| 18.1 kDa class I heat shock protein | Mature endosperm | 1-D |
| Translationally controlled tumor protein homolog | Mature endosperm | 1-D |
| ADP, ATP carrier protein, mitochondrial | Mature endosperm, embryo | 1-D |
| GTP-binding nuclear protein Ran/TC4 | Mature endosperm, embryo | 1-D |
| Putative membrane protein ycf1 | Mature endosperm | 1-D |
| Ribulose bisphosphate carboxylase large chain | Immature endosperm, embryo | 1-D |
| Chaperonin 60 subunit alpha 1, chloroplastic | Immature endosperm | 1-D |
| Histone H2A | Immature endosperm | 1-D |
| Histone H2B | Immature endosperm | 1-D |
| Poly [ADP-ribose] polymerase 3 | Immature endosperm | 1-D |
| Chaperone protein ClpC1 | Immature endosperm, embryo | 1-D |
| 17.5 kDa class I heat shock protein | Immature endosperm | 1-D |
| WPP domain-interacting tail-anchored protein 2 | Immature endosperm | 1-D |
| Probable receptor-like protein kinase At1g49730 | Immature endosperm | 1-D |
| Family 5 extracellular solute-binding protein | Mature endosperm | 2-D |
| Hypothetical Protein RTG_03126 [Rhodotorula glutinis ATCC 204091] | Mature endosperm | 2-D |
| Cytosolic pyruvate orthophosphate dikinase [Oryza sativa Indica Group] | Mature endosperm | 2-D |
| 35 kDa seed maturation protein [Glycine max] | Mature endosperm | 2-D |
| Hypothetical protein ARALYDRAFT_482642 | Mature endosperm | 2-D |
| Putative Protein phosphatase-2C [Arabidopsis thaliana] | Mature endosperm | 2-D |
| Mitochondrial distribution and morphology protein 12 [Magnaporthe oryzae 70-15] | Mature endosperm | 2-D |
| At1g59500 [Arabidopsis thaliana] | Mature endosperm | 2-D |
| Short chain dehydrogenase, putative [Ricinus communis] | Mature endosperm | 2-D |
| 17.7 kDa heat shock protein [Helianthus annuus] | Mature endosperm | 2-D |
| Phosphoenolpyruvate carboxylase 2 | Embryo | 1-D |
| Clathrin heavy chain 1 | Embryo | 1-D |
| Chaperonin 60 subunit beta 3 | Embryo | 1-D |
| Ras-related protein RABA1d | embryo | 1-D |
| ATP-citrate synthase beta chain protein 1 | Embryo | 1-D |
| Glycine--tRNA ligase 1 | Embryo | 1-D |
| UDP-arabinopyranose mutase 1 | Embryo | 1-D |
| Dihydrolipoyllysine-residue acetyltransferase component 2 of pyruvate dehydrogenase complex | embryo | 1-D |
| Serine/threonine-protein kinase SAPK6 | Embryo | 1-D |
| Alpha-1,4 glucan phosphorylase L-1 isozyme | Embryo | 1-D |
| GTP-binding protein YPTC1 | Embryo | 1-D |
| Callose synthase 11 | embryo | 1-D |
